# Intrathecal synthesis of anti-Hu antibodies distinguishes patients with paraneoplastic peripheral neuropathy and encephalitis

**DOI:** 10.1186/s12883-016-0657-5

**Published:** 2016-08-11

**Authors:** Philipp Schwenkenbecher, Lisa Priya Chacko, Ulrich Wurster, Kaweh Pars, Refik Pul, Kurt-Wolfram Sühs, Martin Stangel, Thomas Skripuletz

**Affiliations:** Department of Neurology, Clinical Neuroimmunology and Neurochemistry, Hannover Medical School, Carl-Neuberg-Str-1, 30625 Hanover, Germany

## Abstract

**Background:**

Paraneoplastic syndromes are serious immune caused diseases of the peripheral and/or central nervous system associated with malignant neoplasm. Symptoms develop when antibodies against antigens expressed by tumor cells cross-react with neuronal proteins. Antineuronal antibodies are usually examined in patient’s sera while examination of the cerebrospinal fluid (CSF) often fails. Furthermore, the few previous reports describing CSF data summarized different antineuronal antibodies and/or regarded patients with different neurological symptoms as one group.

**Methods:**

We retrospectively evaluated data of 18 patients with paraneoplastic syndromes due to anti-Hu antibodies. The study aimed to differentiate patients with peripheral neuropathy and encephalitis by cerebrospinal fluid (CSF) parameters including anti-Hu antibody titers.

**Results:**

Our results confirm previous observations that serum titers of anti-Hu antibodies and standard CSF values do not differ between patients with neuropathy and encephalitis. However, analysis of CSF anti-Hu titers and calculating the intrathecal synthesis helped to discriminate between both groups.

**Conclusion:**

In conclusion, our results indicate that patients even with one defined antineuronal antibody need to be regarded separately depending on the involved location of the nervous system. We recommend incorporation of anti-Hu analyses in the CSF and calculating the intrathecal synthesis in patients with anti-Hu syndrome.

## Background

Paraneoplastic neurological syndromes (PNS) are rare neurological disorders associated with malignant neoplasm [1]. The pathomechanisms are not caused by the tumor itself, metastases, or the appropriate cancer therapy. Instead, an immune-mediated remote cancer effect is considered. It is widely accepted that antibodies against ectopic antigens expressed by tumor cells cross-react with antigens of the nervous system and initiate an immune cascade leading to neurodegeneration [2]. To date, several “well-characterized” anti-neuronal antibodies (Hu, Ri, Yo, CV2/CRMP5, Ma1, Ma2/Ta, and Amphiphysin) were identified. The anti-Hu antibody has been first described in 1985 in patients with small cell lung cancer (SCLC) and sub-acute sensory neuropathy [3] and has turned out as the most frequent paraneoplastic antibody [4]. The clinical spectrum of paraneoplastic anti-Hu syndromes is broad and different stages and syndromes such as encephalomyelitis, limbic encephalitis, subacute cerebellar degeneration, and sensory neuropathy have been described [5]. The term encephalomyelitis was introduced to define patients with cancer and neurological symptoms including the whole nervous system. However, this rather general term needs improvement since it does not describe patient’s key clinical symptoms. It was thus recommended by others to describe the paraneoplastic disorder according to the leading focal syndrome that involves best patients signs and symptoms e.g. brainstem encephalitis [5].

CSF analysis is crucial in the diagnosis of nervous system infections and inflammatory driven autoimmune diseases. CSF results such as cell count, glucose, total protein, and oligoclonal bands were previously reported in few series of patients with anti-Hu associated paraneoplastic syndromes [4]. Anti-Hu antibodies are usually identified in patients sera while only few studies have, to date, evaluated CSF titers and/or intrathecal synthesis of anti-Hu antibodies [6, 7]. The literature still lacks detailed CSF studies in patients with paraneoplastic anti-Hu syndromes. Here, we performed a thorough evaluation of clinical and laboratory data with special interest on CSF. The aim of our study was to examine CSF changes and intrathecal synthesis of anti-Hu antibodies in patients with paraneoplastic peripheral neuropathy in comparison to patients with paraneoplastic inflammation of the central nervous system.

## Methods

### Patients

The retrospectively evaluated data originate from 25 patients collected for routine diagnosis in the Hannover Medical School in the time from 1996 to 2015. Only patients who fulfilled the diagnostic criteria of a paraneoplastic syndrome and were tested positive for anti-Hu antibodies in serum by two independent analytical techniques were included in the study. CSF was obtained in all of these patients. The investigation was approved by the local ethics committee of the Hannover Medical School.

### CSF and serum analytical procedures

CSF and serum were analysed by routine methods [8]. CSF leukocytes were counted manually with a Fuchs-Rosenthal counting chamber. CSF total protein was determined by the Bradford dye-binding procedure. IgG, IgA, IgM, and albumin were measured in CSF and serum in the same latex enhanced assay by kinetic nephelometry (Beckman Coulter IMMAGE). CSF-serum quotients of IgG, IgA, IgM, and albumin were calculated [9]. The function of the blood–CSF barrier was estimated by CSF-serum albumin quotients (QAlb). The age-adapted upper limit of QAlb was calculated using the formula QAlb = 4 + (age in years/15) [9]. Intrathecal synthesis of IgG, IgA, and IgM was calculated based on the method of Reiber-Felgenhauer referring the IgG, IgA, and IgM quotients to the albumin quotient [9]. CSF oligoclonal bands (OCB) were determined by isoelectric focusing in polyacrylamide gels with consecutive silver staining. All analyses were performed in the neuroimmunological lab which participates in external quality control programs [10].

Anti-Hu antibodies were detected by indirect immunohistochemistry using commercially available cerebellum primate slides (INOVA Diagnostics) and immunoblots with recombinant antigens (PNS-Blot, Ravo Diagnostika) according to the instructions of the manufacturers. Anti-Hu antibody titration was performed by indirect immunohistochemistry using cerebellum primate slides with increasing dilutions. The starting dilution was 1:100 for serum and 1:1 for CSF. To obtain an intrathecal synthesis of anti-Hu antibodies the same approach was used to calculate an antibody index (AI) in CNS inflammatory/infectious diseases [11]. Anti-Hu IgG antibody specific index (AI) was calculated according to the formula (CSF anti-Hu IgG/serum anti-Hu IgG)/(CSF IgG total/serum IgG total) [12]. In case of intrathecal synthesis of IgG the following formula was used: (CSF anti-Hu IgG/serum anti-Hu IgG)/Qlim. Qlim represents the IgG fraction in CSF originating only from blood and was calculated according to the individual’s albumin quotient [12]. AI values > 2.0 indicate specific antibody synthesis in the CNS.

### Statistical analysis

Fisher’s exact test was performed to assess the association between dichotomous variables. For comparison of two independent groups, the two-tailed unpaired two-sided Mann–Whitney test was performed. For each comparison, a *P* value <0.05 was considered as statistically significant.

## Results

### Patient’s characteristics

A total of 25 patients with neurological symptoms confirmative with a paraneoplastic syndrome were tested positive for anti-Hu antibodies in serum by using the immunoblot technique. Eighteen patients showed a clear intense band while in seven patients only a borderline or moderate intense band was found. By using immunohistochemistry none of the latter seven patients showed an anti-neuronal staining in the cerebellar tissue, and thus, these patients were considered negative for anti-Hu antibodies. The weak positive immunoblot band was rather the result of a non-specific cross-reaction. In three of these patients concurrent autoimmunological diseases were known (systemic lupus erythematosus, Sjoegren’s syndrome, neurosarcoidosis). Another patient presented with acute lymphatic leukemia.

**Fig. 1:**
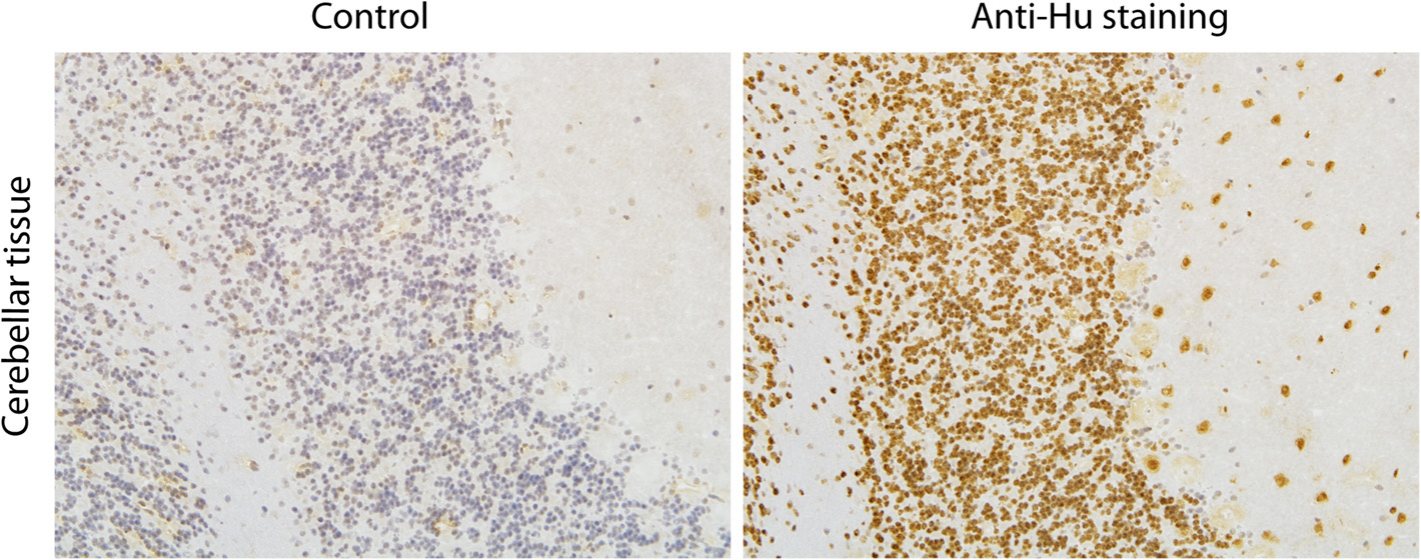
Anti-Hu staining in the primate cerebellum as demonstrated by immunohistochemistry. Cerebellar tissues were either incubated with serum of a control healthy patient (left) or with serum of a patient with a paraneoplastic syndrome due to anti-Hu antibodies

The remaining 18 patients with intense blot results presented a clear anti-neuronal staining in the cerebellar tissue. The typical staining for anti-Hu antibodies showed a nuclear staining of neurons in the granular, purkinje, and molecular cell layer (Fig. 1). Only these patients were considered positive for anti-Hu antibodies and were included in our study. Patients were categorized into two groups according to the location of nervous system affection as identified by symptoms and clinical signs leading to hospitalization: isolated peripheral neuropathy or encephalitis. Patients with encephalitis were further divided into two groups: rhombencephalitis or limbic encephalitis (Table 1). The cohort consisted of eleven females and seven men. The median age of all patients was 61 years with a range from 48 to 76 years.

**Table 1:** Patient’s characteristics

Characteristics	Females (number)	Age (years)	Duration of symptoms (months)	Symptoms preceeding malignancy (number)	Malignancy	
					All tumors (number)	Lung cancer (number)
All patients	11/18	61 (48–76)	4 (1–36)	15/18	16/18	14/18
Peripheral neuropathy	5/8	59 (54–76)	3 (1–36)	7/8	7/8	5/8
Encephalitis	6/10	61 (48–74)	4 (1–15)	8/10	9/10	9/10
Rhombencephalitis	3/7	61 (48–73)	13 (3–15)	6/7	6/7	6/7
Limbic encephalitis	3/3	55, 61, 74	1, 3, 4	2/3	3/3	3/3
*p value*	1.0	0.6	0.5	1.0	1.0	0.3

Age and duration of symptoms until diagnosis are presented by median with lowest and highest values. *P* values indicate comparison between peripheral neuropathy and encephalitis

### Peripheral neuropathy associated with anti-Hu antibodies

In our cohort, eight patients showed peripheral neuropathy without signs of central nervous system (CNS) involvement (Table 1). Duration of symptoms until the diagnosis of anti-Hu syndrome was less than six months in five patients and longer than 12 months in only one patient.

At first presentation in our clinic, all eight patients reported sensory abnormalities in terms of numbness and tingle paresthesia. Involvement of both hands and feet was found in five patients. In two patients, symptoms were initially restricted to either hands or feet but extended to all extremities in the course of the disease. One patient reported sensory deficits of both feet only which remained localized in the lower extremities. At first examination weakness of extremities was not found, but during the disease course, four patients developed distal arm and leg palsy. Signs of sensory ataxia were found in six patients at first presentation but all patients developed relevant gait disturbance in the course of disease.

Electrophysiological examinations revealed axonal damage in all patients, while four patients showed signs of an additional demyelinating damage. In all patients sensory nerves were affected. Four patients showed an additional damage of motor nerves.

### Encephalitis associated with anti-Hu antibodies

#### Rhombencephalitis

In seven patients involvement of the rhombencephalon defined as either cerebellar or brainstem symptoms was found (Table 1). The duration from first neurological symptom until diagnosis of anti-Hu syndrome was less than six months in three patients and more than 12 months in four patients.

Four patients suffered from cerebellar and brainstem symptoms including cerebellar ataxia, scanning dysarthria, dysphagia, hemiparesis, oculomotor disturbance (abducens palsy, internuclear opthalmoplegia), saccadic eye movement, and/or nystagmus. In three patients brainstem symptoms without cerebellar involvement were found including oculomotor disturbance (abducens palsy), hemiparesis, and hemihypesthesia. One patient with brainstem symptoms reported numbness on the left side of the face, on the left arm and leg. In this patient, MRI showed a cervical lesion indicating an additional myelitis.

Only one patient presented with symptoms of isolated CNS involvement. The other six patients reported numbness and tingle paresthesia of hands and feet as first symptoms. In these patients a concomitant peripheral neuropathy was found. In the disease course these patients developed an additional distal palsy of hands and feet.

#### Limbic encephalitis

Limbic encephalitis was diagnosed in three patients (Table 1). The duration from onset of neurological symptoms until diagnosis of a paraneoplastic syndrome ranged from 1 to 4 months. Symptoms included aggressive behavior, anxiety, disorientation, and a mnestic syndrome. Two patients suffered from focal epileptic seizures. In both patients EEG showed epileptic foci (right hemisphere or parietal left). One patient developed general seizures and status epilepticus occurred three times during the disease course. Only one patient suffered from an additional sensory neuropathy before onset of encephalitis.

### CSF findings in peripheral neuropathy

**Fig. 2:**
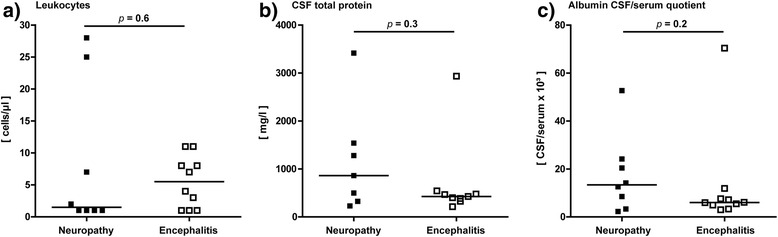
Cerebrospinal fluid results in patients with anti-Hu syndrome. Graphs show the distribution of cell count (**a**), total protein (**b**), and albumin CSF/serum quotients (**c**) in patients with peripheral neuropathy and encephalitis. The bars represent the median values in each group

Three patients showed a slightly elevated CSF cell count between 7 and 28 cells/μl, while in five patients normal cell counts were found (Table 2, Fig. 2). CSF lactate concentrations were normal in all patients. Total protein was increased in four patients. Measurements of QAlb, which is the best indicator for a blood-CSF barrier dysfunction, revealed age-corrected increased values in six patients. Barrier impairment was severe in 3 patients (QAlb >20) and mild to moderate in the other three patients.

**Table 2:** Cerebrospinal fluid laboratory findings

Characteristics	Pleocytosis (≥5 cells/μl)	Protein (>500 mg/l)	Blood-CSF-barrier dysfunction	Intrathecal synthesis			CSF oligoclonal bands
				IgG	IgM	IgA	
All patients	8/18	6/18	8/18	6/18	2/18	2/18	14/18
Peripheral neuropathy	3/8	4/8	6/8	1/8	1/8	0/8	3/8
Encephalitis	5/10	2/10	2/10	5/10	1/10	2/10	10/10
Rhombencephalitis	4/7	1/7	1/7	3/7	1/10	1/7	7/7
Limbic encephalitis	1/3	1/3	1/3	2/3	0/3	1/3	3/3
*p value*	0.7	0.3	0.1	0.2	1.0	0.5	0.01

*P* values indicate comparison between peripheral neuropathy and encephalitis

Oligoclonal bands restricted to the CSF were found in only three patients (38 %) indicating intrathecal IgG synthesis. One of these patients showed a combination of oligoclonal bands restricted to the CSF and identical oligoclonal IgG bands in CSF and serum (type 3). Identical oligoclonal IgG bands in CSF and serum were found in three patients with peripheral neuropathy. Intrathecal synthesis of immunoglobulins of either IgM, or IgG, or IgA occurred in only two patients. Intrathecal synthesis of IgM as calculated by the method of Reiber-Felgenhauer was found in one patient and IgG synthesis in another one patient. Both patients presented oligoclonal bands restricted to the CSF.

### CSF findings in encephalitis

Five patients with encephalitis showed a slightly elevated cell count between 7 and 11 cells/μl, while in the other five patients normal cell counts were found (Table 2, Fig. 2). All patients had normal lactate concentrations. Total protein was increased in two patients. A blood-CSF barrier dysfunction measured by QAlb was detected in these two patients. Barrier impairment was severe in one patient (QAlb >20) and mild in the other patient.

Oligoclonal bands restricted to the CSF were found in all ten patients (100 %) indicating intrathecal IgG synthesis. Three of these patients showed a combination of oligoclonal bands restricted to the CSF and identical oligoclonal IgG bands in CSF and serum (type 3). Intrathecal synthesis of immunoglobulins of either IgM, or IgG, or IgA occurred in five patients. One of these patients presented with a combined three-class reaction of intrathecal synthesis of IgG, IgM, and IgA. One patient showed a two-class reaction of intrathecal synthesis of IgG and IgA. The remaining three patients showed an isolated IgG synthesis.

### Anti-Hu antibody titers in serum and CSF

**Fig. 3:**
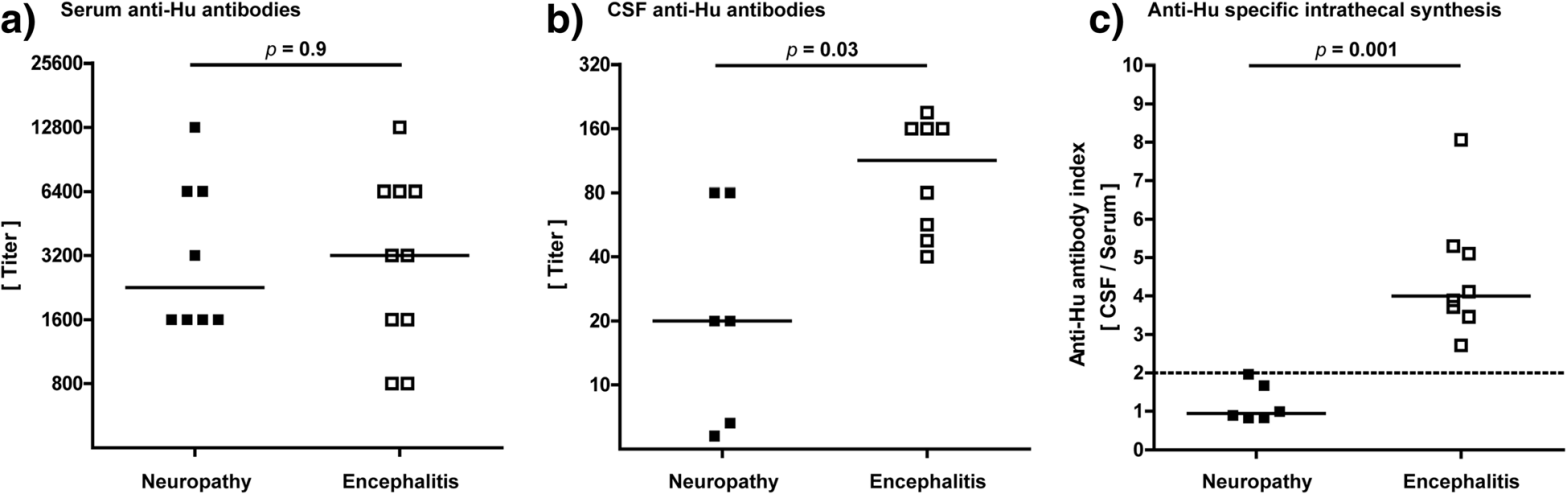
Serum and cerebrospinal fluid anti-Hu titers. In **a**, distribution of serum anti-Hu titers in patients with peripheral neuropathy and encephalitis is shown. In **b**, anti-Hu titers are shown in the cerebrospinal fluid. In **c**, antibody index of anti-Hu specific IgG is shown which indicates specific intrathecal antibody synthesis. Antigen index values >2.0 indicate an intrathecal synthesis

Serum anti-Hu titers were available from all 18 patients and were similar in patients with peripheral neuropathy (between 1:1600 and 1:12.800) and patients with CNS involvement (1:800 and 1:12.800; Fig. 3). CSF anti-Hu titers were available from 6/8 patients with neuropathy and from 8/10 patients with encephalitis. As shown in Fig. 3, anti-Hu titers in the CSF tended to lower values in patients with peripheral neuropathy (between 1:1 and 1:80) as compared to patients with encephalitis (between 1:40 and 1:200).

The antibody index (AI) was calculated with the aim to indicate an intrathecal synthesis of anti-Hu antibodies. Intrathecal synthesis of anti-Hu antibodies was found in all eight patients with encephalitis (Fig. 3). In contrast, in patients with peripheral neuropathy intrathecal synthesis of anti-Hu antibodies (>2) did not occur. However, two patients showed borderline values (AI of 1.67 and 1.97).

### Neuroimaging findings

Cranial MRI confirmed the diagnosis of limbic encephalitis in one patient showing T2 weighted lesions in both temporal lobes. In one patient with rhombencephalitis MRI depicted a T2 weighted lesion in the spinal cord. In the other two patients with limbic encephalitis and all seven patients with brainstem encephalitis MRI did not reveal radiological sings of inflammation in the brain. In these patients, only signs of brain atrophy and unspecific vascular lesions unrelated to a paraneoplastic disease were found. Cerebral metastases and leptomeningeal enhancement as a sign of neoplastic meningitis were neither detected in patients with rhombencephalitis nor limbic encephalitis.

### Malignancy

In 16 patients (89 %) with anti-Hu syndrome a malignant tumor was either known or diagnosed consecutively (Table 1). In three patients an underlying malignancy was known before the diagnosis of anti-Hu syndrome. In two of these patients lung cancer (SCLC and non-small cell lung cancer (NSCLC)) was diagnosed 1 month before, while another patient suffered from breast cancer since 5 months.

After diagnosis of anti-Hu syndrome, tumor screening revealed new lung or breast malignancy in 13 patients. Histological analyses revealed SCLC in ten patients, NSCLC in two patients, and breast cancer in one patient. In eight patients new malignancy was found already at the time of initial hospitalization due to anti-Hu syndrome. In four patients tumor was found within 12 months, while in one patient tumor was found after 5 years. Two patients with newly diagnosed NSCLC had a positive history of breast cancer. Breast cancer was found 4 and 9 years before, respectively, and was considered to be in remission in both patients.

In one patient malignancy was not found at the time point of anti-Hu syndrome and patient did not return for follow up. In another one patient malignancy was not found within a follow-up of 20 months.

Metastases were found in one patient with lung cancer (in the ribs) and one patient with breast cancer (liver). In the other patients no metastases were found.

## Discussion

In this retrospective study, we analyzed characteristics of clinical and immunological features in patients with paraneoplastic syndromes associated with anti-Hu antibodies. Our results show crucial differences in CSF results depending on whether the peripheral or central nervous system is affected. We found that intrathecal synthesis of anti-Hu antibodies discriminates between patients with isolated peripheral neuropathy and patients with encephalitis.

It is widely accepted that CSF analysis is essential in the diagnosis of paraneoplastic syndromes [5]. However, there is only few data regarding CSF results in patients with anti-Hu syndromes. Indeed, one large study included 170 patients from the PNS European database diagnosed in Europe after 2000 [4]. However, CSF results are incomplete and oligoclonal bands were studied in 73 patients only (43 %). Furthermore, patient’s information is lacking and patients with anti-Hu syndrome were regarded as one group. Thus, the interpretation of oligoclonal bands (positive in 59 % of these patients) underlies severe limitations. The main limitation of other reports is the fact that paraneoplastic syndromes with different anti-neuronal antibodies were summarized and/or regarded as one group [13, 14]. Here, we show that even in patients with one defined antineuronal antibody immunological results need to be interpreted separately depending on the involved location of the nervous system. In our cohort patients suffered either from isolated peripheral neuropathy or encephalitis (rhombencephalitis or limbic encephalitis). CSF analysis revealed oligoclonal bands indicating intrathecal IgG synthesis in all ten patients with encephalitis associated with anti-Hu antibodies (100 %). In contrast, in patients with peripheral neuropathy oligoclonal bands were found infrequently (38 %). CSF standard parameters such as cell count and blood-CSF barrier dysfunction (albumin ratio, total protein) were similarly distributed in both groups and do not help to differentiate between them.

It is known that serum titers of anti-Hu antibodies do not correlate with disease progression. In our work, we confirm these previous observations as we found similar serum titers in patients with peripheral and central nervous system involvement. In contrast, all patients with encephalitis displayed an intrathecal synthesis of anti-Hu antibodies, while patients with peripheral neuropathy did not. We hypothesize that these results indicate different disease progression in isolated peripheral neuropathy and encephalitis. It is well known that anti-Hu antibodies react with nuclei of both peripheral and central nervous system neurons [15]. In our cohort, in the majority of patients with encephalitis the paraneoplastic syndrome started with peripheral sensory neuropathy. Since the malignancy is found in the periphery outside the CNS it seems to be obvious that anti-Hu antibodies first react with peripheral neurons of the dorsal root ganglia resulting in peripheral sensory neuropathy. During the disease course in some patients plasma cells within the CNS start to produce anti-Hu antibodies as well (which is measured as intrathecal synthesis of this antibody) consequently resulting in encephalitis. The pathomechanisms of this crossover are not known. We can only speculate that peripheral antibodies passively diffuse into the CNS and then react with neurons. This reaction might initiate an immune cascade in which local B cells differentiate into plasma cells and produce anti-Hu antibodies locally in the CNS. However, this crossover does not occur in all patients. It was described that some patients with peripheral neuropathy due to anti-Hu antibodies do not develop encephalitis [16].

Neuropathological brain studies of patients with anti-Hu encephalitis revealed severe loss of neurons in combination with inflammatory infiltrates with predominantly T lymphocytes [17]. These inflammatory signs and neurodegeneration suggest that early efficient treatment might be beneficial for the clinical outcome. We hypothesize that the treatment benefit might be of particular success in patients with isolated peripheral neuropathy prior to additional encephalitis. Analysis of the CSF with determination of intrathecal synthesis of anti-Hu antibodies presents a useful tool to discriminate between these both groups of patients with and without involvement of the CNS. Our findings might be thus relevant for the treatment of these patients.

## Conclusion

Our data show that patients with paraneoplastic syndromes due to anti-Hu antibodies need to be regarded separately depending on the involvement of the peripheral or central nervous system. CSF analysis with determination of intrathecal synthesis of anti-Hu antibodies presents an important tool to discriminate these patients. However, with respect to the limited group size a larger multicenter study is needed to confirm our results.

## Abbreviations

CNS, central nervous system; CSF, cerebrospinal fluid; MRI, magnetic resonance imaging; NSCLC, non-small cell lung cancer; OCB, CSF-specific oligoclonal bands; PNS, paraneoplastic neurological syndromes; QAlb, CSF-serum albumin quotients; SCLC, small cell lung cancer
